# Wearable Robotic Glove Design Using Surface-Mounted Actuators

**DOI:** 10.3389/fbioe.2020.548947

**Published:** 2020-09-25

**Authors:** Jaeyoung Park, Inchan Hwang, Woochan Lee

**Affiliations:** ^1^Robotics and Media Institute, Korea Institute of Science and Technology, Seoul, South Korea; ^2^Department of Mechanical Engineering, Seoul National University, Seoul, South Korea; ^3^Department of Electrical Engineering, Incheon National University, Incheon, South Korea

**Keywords:** robotic hand, soft robotics, biomimetic, surface-mounted actuator, anthropomorphic hand

## Abstract

We propose a novel wearable robotic glove or exo-glove design scalable to the variation of the hand kinematics. While most of the traditional robot hand is driven by rotating the joint directly with a rigid body, our exo-glove deforms a robotic finger's skin and, thus, the hand skeleton joints. Multiple tendons woven on the exo-glove's surface can make multi-DOF finger joint motions. We allocated tendons to mimic a hand's intrinsic and extrinsic muscles. Thus, a robotic hand actuated with the exo-glove can perform natural finger motions, including abduction/adduction and flexion/extension of finger joints. Moreover, additional tendons for the thumb enable power grips and the robotic hand's human-like motion. The proposed design approach places all the actuators on the surface without directly actuating any of the hand skeleton's joint. Therefore, a random hand skeleton can work as a robotic hand by putting the wearable robotic glove on it. Thus, the proposed model provides a high degree of freedom on choosing hand skeletons. We expect the aforementioned biomimetic features of our proposed method will benefit not only traditional robotic hands design but also the design of prosthetic hands and robot power-assisted hand glove.

## 1. Introduction

A robotic hand imposes significant constraints on the manipulability of a robotic system so that its ability often decides the type and the complexity of a task. In this context, an anthropomorphic robotic hand has been a popular research subject in robot manipulation area, considering the dexterity of the human hand. Most of the previously proposed anthropomorphic robotic hands have been developed to mimic the human hand's ability in terms of motion and tactile sensitivity (Kawasaki et al., 2002; Gama Melo et al., 2014). Many of the robotic hands could imitate a human hand' grasping motion, as suggested by Cutkosky (1989). Also, former researches on anthropomorphic hand demonstrated that the hand could manipulate an object so that its performance is similar to that of a human hand's dexterity (Lee et al., 2016; Xu and Todorov, 2016; Jeong et al., 2017).

Most of the previously developed anthropomorphic hands have been implemented in the form of typical rigid body robotic linkages, which has the advantage of sturdy structure and the ease of position and force control. On the other hand, there are major robot applications requiring lightweight and useability by a human user, such as a prosthetic hand and a haptic master glove. In this case, the aforementioned rigid body approach may not be an optimal solution due to its relatively heavyweight and less adaptable or scalable nature.

The use of artificial tendon is a classical approach in robot hand design due to its advantage in lightweight of the end-effector. Thus, researchers in robotics have studied the tendon-driven hand in various aspects, including mechanism, control strategy, and stability (Lee et al., 1994; Jacobsen et al., 1998; Kobayashi et al., 1998). Regarding the anthropomorphic robotic hand, the German Aerospace Center (DLR) has developed efficient anthropomorphic robotics hand-arm systems and demonstrated the efficient performance of their system (Butterfa et al., 2001; Grebenstein et al., 2011). Also, Deshpande et al. (2008) proposed an anthropomorphic robotic hand system called the anatomically correct testbed (ACT) hand. The robotic hand mimics the structures of the human hand's muscle and skeleton to replicate the human hand motion and force (Deshpande et al., 2008; Niehues and Deshpande, 2017). Also, Kim et al. recently proposed an anthropomorphic robotic hand design driven with tendon (Kim et al., 2019). They demonstrated that their proposed robotic hand could repeat precision finger motion under various impacts. The researches mentioned above successfully implemented anthropomorphic hands that could naturally emulate diverse human hand postures. Most of them were implemented for a rigid structure, which has the advantage of correct control of motion and desired contact force. However, such a characteristic restricts the flexibility in the system scalability, in terms of the robot hand size, finger shape, finger length, the number of fingers, etc. Also, having a fixed robot hand size means the restriction of applications, including a prosthetic hand.

A wearable robotic hand is one of the less-studied form factor compared to other types of anthropomorphic robotic hands. Most of the wearable robotic hands have been developed in the form of an exoskeleton hand interface to be worn on a human user's hand (Sarac et al., 2019). The design of wearable hand interfaces varies by its applications, including the rehabilitations (Kim et al., 2017; Gasser et al., 2019), power assists (Hasegawa et al., 2019), and the haptic interface for virtual interaction (Choi et al., 2016; Son and Park, 2018). The exoskeletal robotic hand has the advantage of the accurate force and position control, but its rigid structure often increases the size and mass of the system. To overcome the limitations of the exoskeletal robotic hand, robotics researchers have developed the soft robotic hand, which consists of deformable structures and materials such as fluids and elastomers (Majidi, 2014). A typical soft robot hand is in the form of a glove with elastic power transmission (Jeong et al., 2013; In et al., 2015). A soft robotic hand has the strength of compact and light design, but it has a weakness in the force display compared to the rigid exoskeletal robotic hand. Thus, the applications of the soft robotic hand have limited to the rehabilitation and haptic interface.

In this paper, we propose a wearable robotic glove (or exo-glove) design worn on a robotic hand skeleton with the actuators mounted on the surface of a robot or human-operator arm. The exo-glove has two layers of tendons, which mimic the functionalities of a human hand's intrinsic and extrinsic muscles (Raszewski and Varacallo, 2018; Dawson-Amoah and Varacallo, 2019). We noted that a typical robotic hand has actuators inside the palm or a finger, which gives constraints on their torque, lowering the manipulator performance. In contrast, the use of external tendons can prevent the power limitation problem by placing actuators behind the manipulator part, but it increases the overall size of the manipulator (Deshpande et al., 2008; Xu and Todorov, 2016). The size issue can be critical for wearable robot applications such as a prosthetic robotic hand or a rehabilitation glove. Notably, installing a prosthetic robotic hand to a partial hand amputee pauses the problems of both limited torque and the limited space for the installation. In this context, our proposed exo-glove is designed with a new tendon-driven mechanism while preventing the problem of increased overall length due to the actuators. Thus, our study's first objective is proposing an exo-glove design with actuators/sensors mounted on the surface of the arm. When installed on a robotic skeleton, it should be able to conduct tasks required for an anthropomorphic robotic hand. On the other hand, the previous studies on wearable robotic glove have focused on evaluating its functionalities as a power assisting or rehabilitation device, assuming to be worn on a human hand (Kang et al., 2016; Yun et al., 2017). In this regard, the second objective of the present study is to evaluate a robotic hand actuated with our proposed exo-glove with tests required for a robotic hand. First, we assess the robotic hand with standard grasp performance test. Then, the robotic hand design is evaluated in three categories; (i) *manipulability*, (ii) *repeatability of motion*, and (iii) *gripping performance*. The robotic hand's manipulability is assessed by posing the robotic hand for various object-gripping tasks, as suggested by Cutkosky (1989). The repeatability of motion is evaluated by tracking five finger motions. Also, we tested the gripping performance by checking the hand's payload with different gripping strategies.

The rest of the paper is organized as follows. First, we illustrate the design of an exo-glove and control strategy. Then, we present the results of evaluating the robotic hand actuated with the exo-glove, for three different performance indices a standard grasp performance test. Finally, we conclude the paper by summarizing the work and mentioning on future work.

## 2. Design of the Wearable Robotic Glove with Skin-Mounted Actuators

In this section, we describe the design of the wearable robotic glove with skin-mounted actuators. We first identify the design requirements of the wearable robotic glove. Then, we show how the required features are implemented in terms of mechanical design and system control. Besides, we explain the working principle of a tactile sensor used for object manipulation.

### 2.1. Two-Layer Design of the Skin-Mounted Actuator Robotic Hand

In this section, we set several constraints on the design of the wearable robotic glove for a robot hand. First, (a) it needs to be able to *(a) move five fingers independently* and *(b) mimic the functionalities of human hand motion*, considering its application as a prosthetic hand. Next, the actuators of the tendon-driven mechanism should *(c) not increase the length of the overall robotic hand*. Since the robotic hand is designed as a wearable robotic hand, the posture of the hand should be estimated with sensors installed on the surface. Finally, the robotic hand should have the *(d) scalability* in increasing/decreasing the motion degree-of-freedom and the ease of changing the actuator.

**Figure 1:**
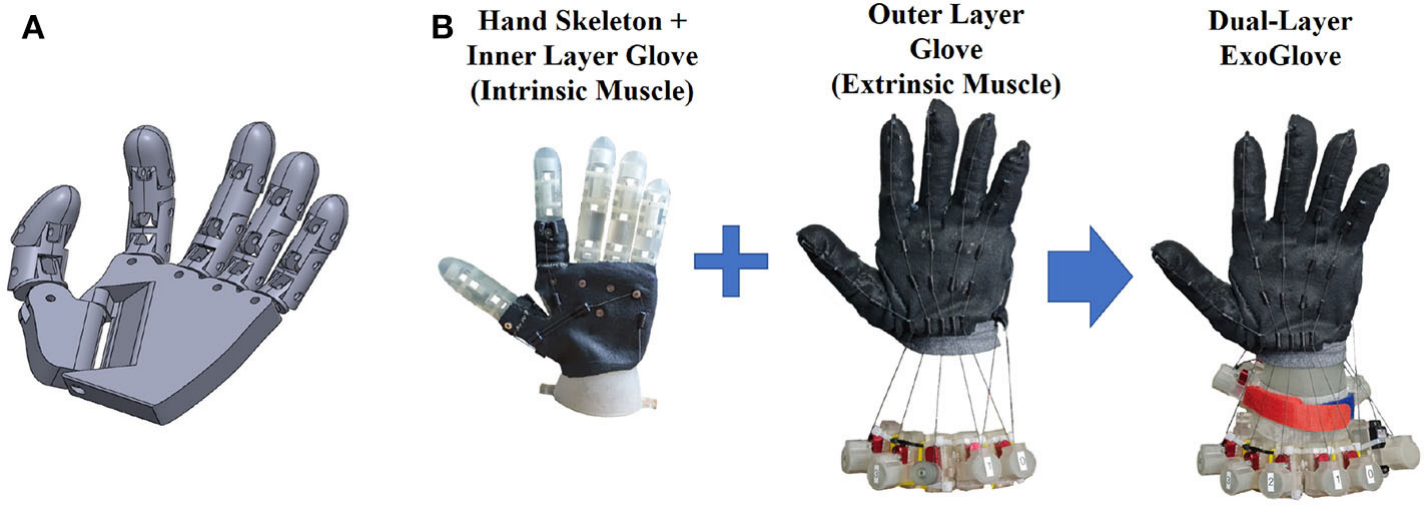
The design and implementation of the robotic hand skeleton in human hand size. **(A)** The mechanical design of the robotic hand skeleton, with full motion degrees of freedom with the size of human hand. **(B)** Assembly of the dual-layer exo-glove robotic hand.

Figure 1 shows the configuration of our proposed dual-layer exo-glove robotic hand. Figure 1A shows the CAD design of a robotic hand's skeleton. It was designed to have as many motion DOF as a human hand, as well as the same dimensionality (166 x 144 x 26 mm). Each finger can strike not only a flexion/extension motion but also an abduction/adduction. In Figure 1B, the assembly of our proposed dual-layer exo-glove robotic hand is explained. An inner-layer exo-glove is installed on the robotic hand skeleton, to mimic the intrinsic hand muscle movement. The outer-layer exo-glove generates the tension force for the extrinsic hand muscle. It is worn outside of the robotic skeleton with the inside-layer exo-glove.

**Figure 2:**
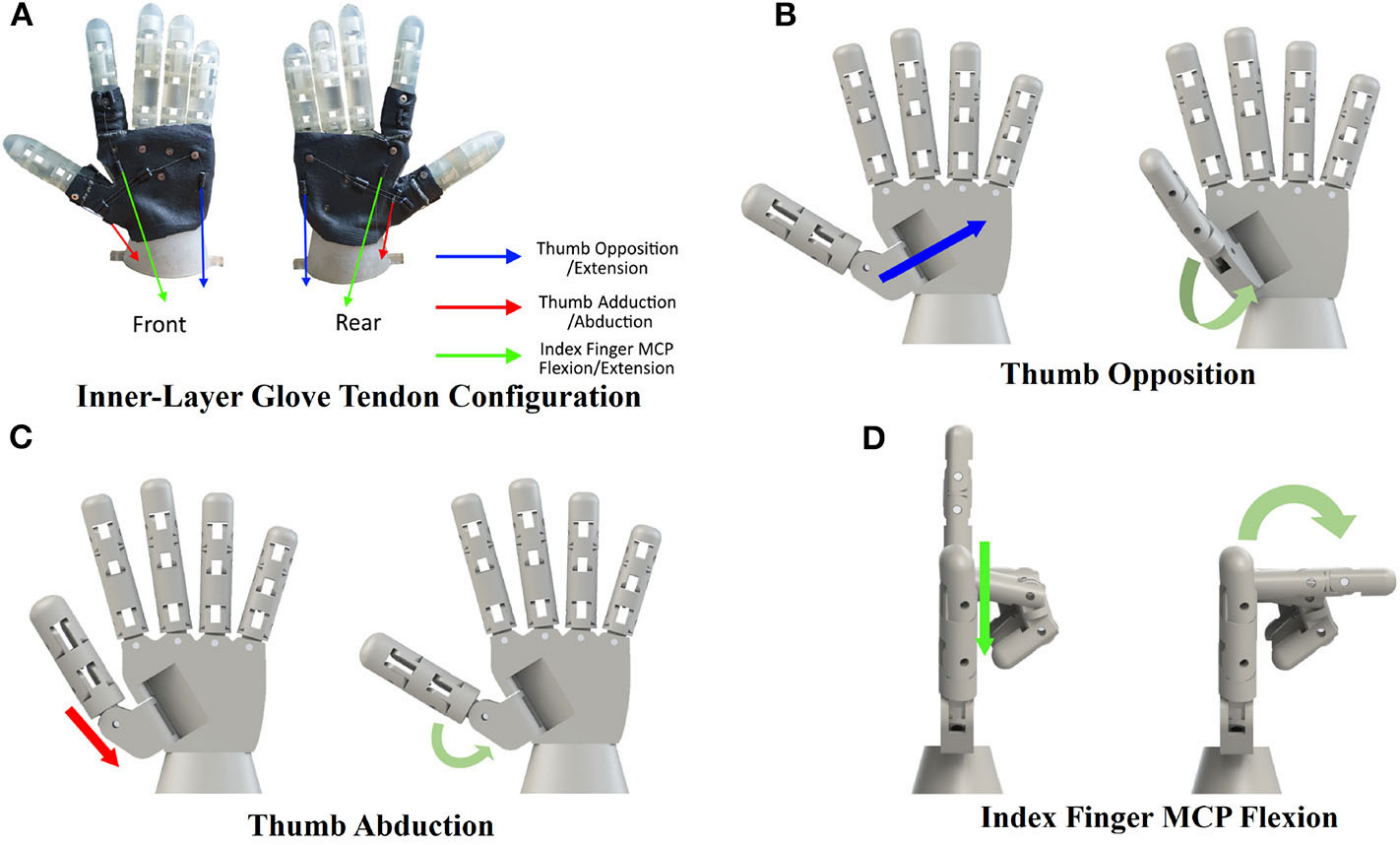
Finger motions invoked by pulling tendons installed on the inside layer of the robot hand. **(A)** The robotic hand skeleton installed with the tendon sheaths for three different finger motions. Each arrow in different colors shows the target motion with a tendon pair. **(B)** Front view of thumb opposition motion by pulling the blue wire in **(A)**. **(C)** Front view of thumb abduction motion by pulling the red wire in **(A)**. **(D)** Side view of index finger's MCP joint flexion by pulling the green wire in **(A)**.

Figure 1A shows the front and rear view of the fabricated robot skeleton with the inside layer of tendon sheaths. We installed the inner-layer for the demonstrate that the wearable mechanism can strike a finger pose that is invoked with intrinsic finger muscles. The tendons were located for three fingers poses, thumb opposition/extension, thumb abduction/adduction, and index-finger metacarpophalangeal (MCP) joint's flexion/extension, which are marked with arrows in different colors in Figure 2A. Figures 2B,D shows finger motions invoked by pulling the tendons installed on the inside layer of the robot hand.

**Figure 3:**
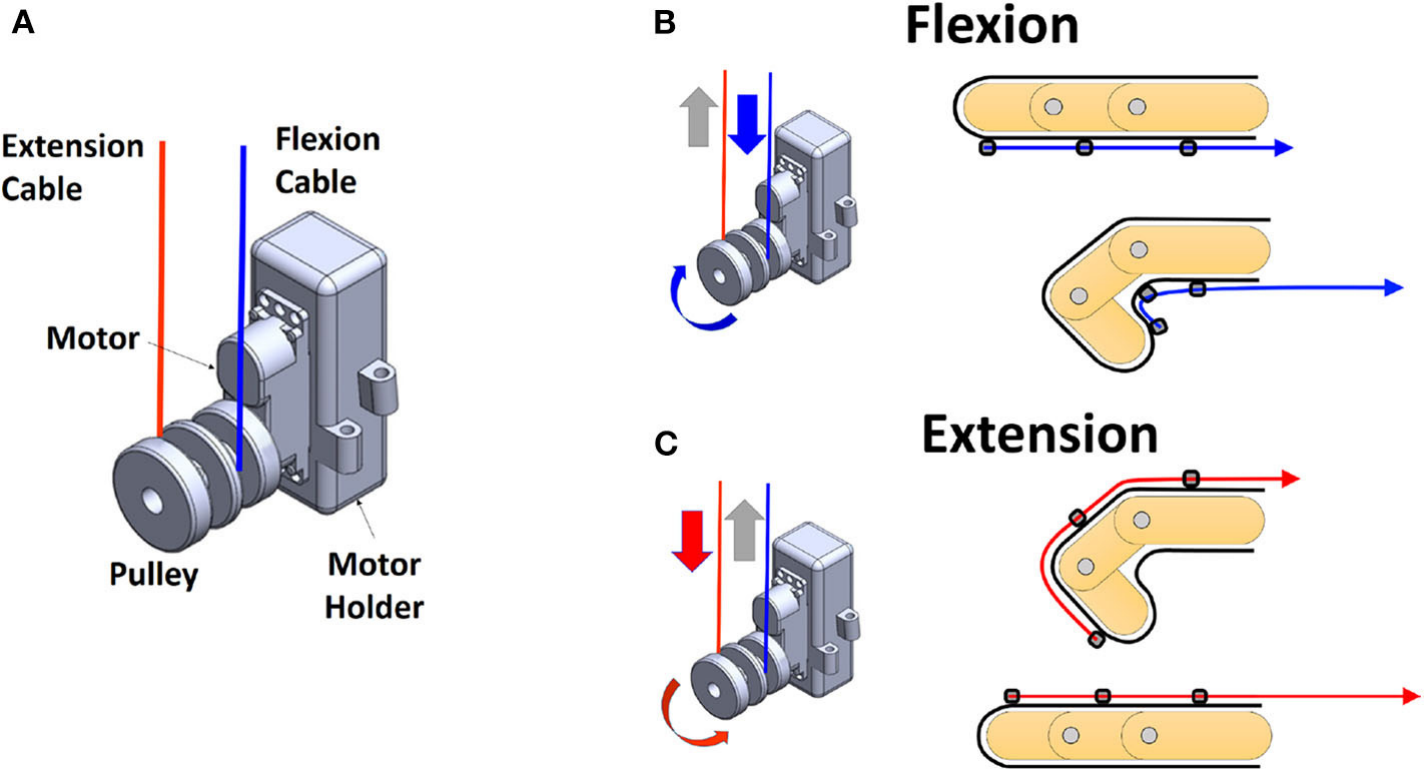
An actuator unit and a finger flexion/extension mechanism with a single motor. **(A)** The overview of the actuator unit. **(B,C)** Schematic drawing of flexion and extension by the rotation direction of the motor.

Figure 3 shows the mechanism of actuating the tendons and thus causes a flexion or extension of a finger. As shown in Figure 3A, an actuator unit consists of a motor (model HV75K, MKS Servo Tech, Ilan, Taiwan), a motor holder, a pulley, and two tendons for flexion and extension of a finger or a joint. The motor holder was designed to be attached to the other one with their hinges interlocked. The two tendons are wound to the pulley in a different direction so that one of the two motions can be switched by changing the direction of the motor. Let us assume that the flexion and extension cables are wound in counter-clockwise and clockwise directions, respectively. Then, if the motor rotates in the clockwise direction, a tension is created for the flexion cable, and the finger flexes as shown on top of Figure 2B. In the meanwhile, the extension cable loosens. Similarly, if the motor rotates in the counter-clockwise direction, a tension is generated for the extension cable so that the finger is extended, as shown in Figure 2B. The actuator units were fabricated in two different sizes to house different types of motors. There are hinge structures on the sides of the motor holder so that the number of the actuator unit can be increased or decreased by the needs of the robotic hand.

**Figure 4:**
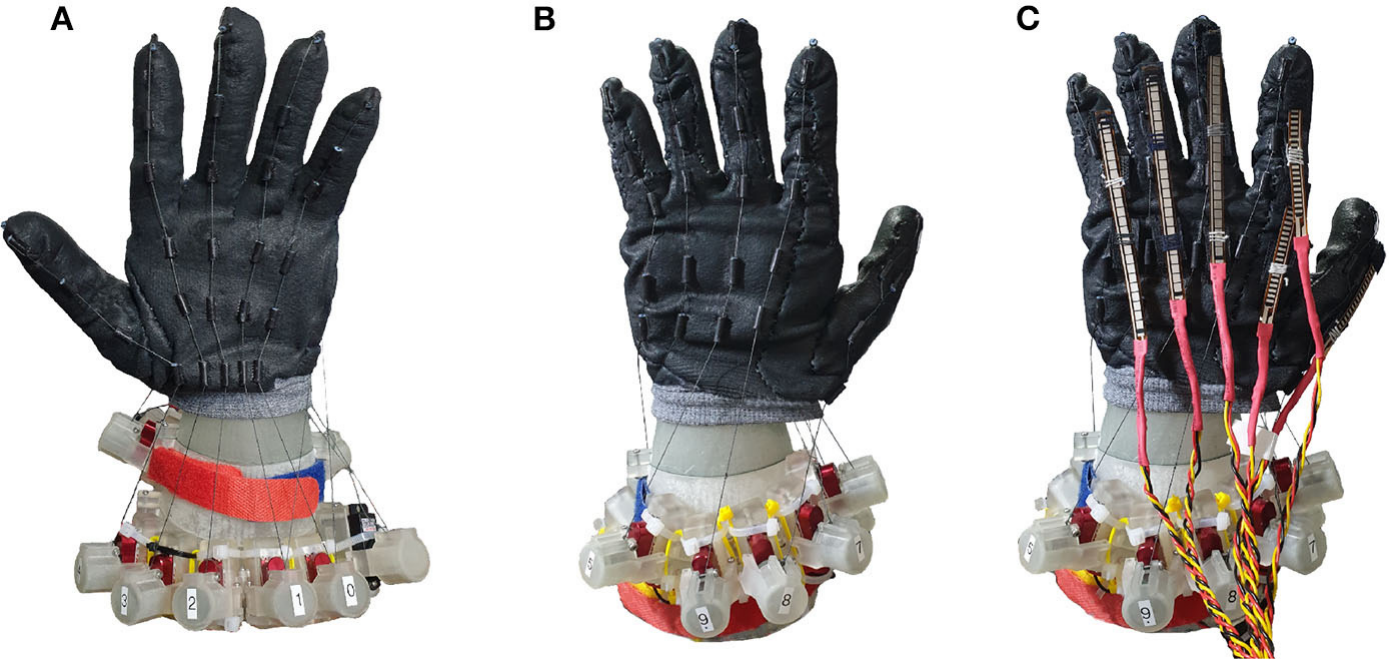
The anthropomorphic robotic hand with the glove for extrinsic muscle and actuators installed. **(A)** The front view of the robotic hand. **(B)** The rear view of the robotic hand. **(C)** The robotic hand with the flex sensors attached for the pose estimation.

**Figure 5:**
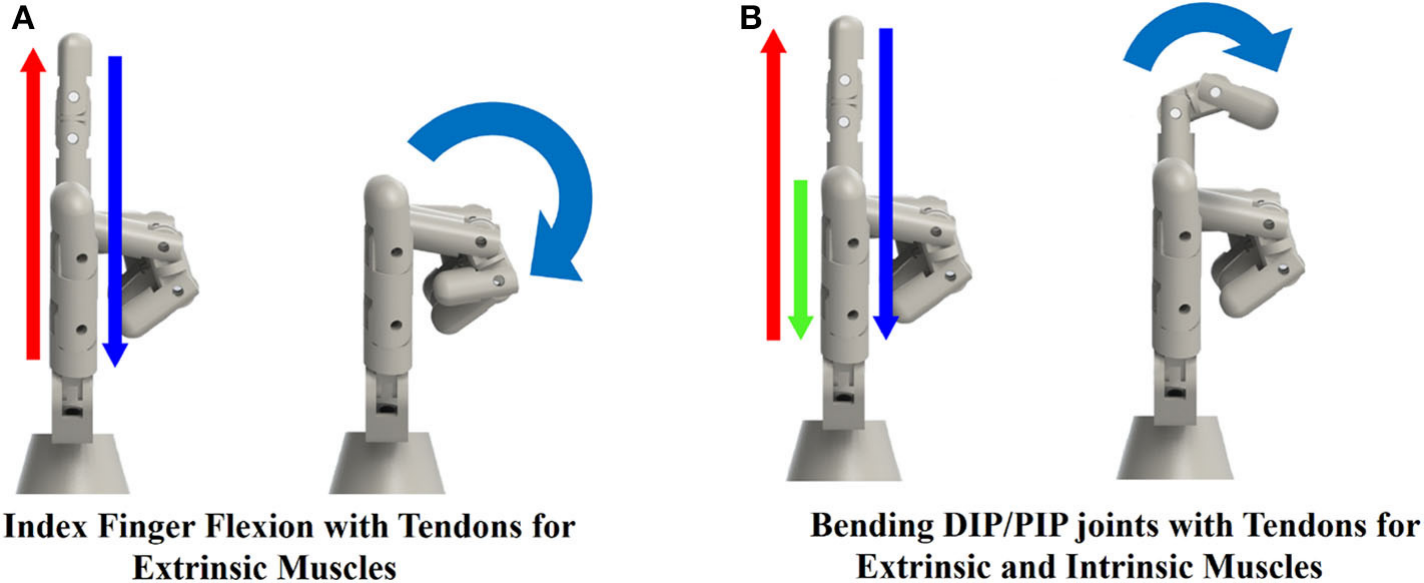
Side view of two different index finger motions. **(A)** Flexion of index finger by pulling the wire on the glove only. All three finger joints bends. **(B)** Flexion of index finger's PIP and DIP joints by pulling the extensor tendon inside, and the flexion tendon on the glove.

We built the dual-layer exo-glove robotic hand by combining the actuator units to a glove with tendon-sheath, worn on the hand skeleton. The glove was made with multiple insulation glove pieces whose surface was nitrile foam coated, which made the glove's surface durable and elastic. The piecewise tendon sheaths were made by gluing inside Teflon tube to outside silicone tube, for the ease of attachment to the glove. We placed the tendon-sheath to mimic the extrinsic muscles on the hand so that each finger can flex and extend with a pair of tendons. Figure 4 shows the robotic hand by combining the glove for extrinsic muscle, actuator units and the hand skeleton. For the thumb, index and middle fingers, two motors were connected to each finger to vary the stiffness of the finger during object manipulation. For low stiffness mode, the flexion tendon on the palm side is pulled while the extension tendon on the backside is loosened. On the contrary, the extension tendon is pulled along with the flexion tendon for high stiffness mode. For the ring finger and little finger, one motor was connected to each finger, considering more active use of the other three fingers for object manipulation. To estimate the finger pose, flex sensor (SparkFun Electronics, CO, USA) were attached to the back of the fingers, as shown in Figure 4C. The dual-layer glove structure can create complex finger motion that cannot be implemented with a single-layer tendon-driven robot exo-glove. Figure 5 shows the example that the index finger can strike complex poses. When the flexion tendon on the outer glove pulls the index finger, all three finger joints bend, as shown in Figure 5A. On the contrary, only PIP and DIP joints can be bent by pulling the flexion tendon on the outside glove and extension tendon on the inside glove. We used a total of eleven actuator units, three for the intrinsic muscle motion, two for each of the thumb/index finger/middle finger flexion and extension, and one for the little finger/ring flexion/extension.

### 2.2. Finger Pose Estimation and Position Control

We aimed to design our robotic exo-glove to have all actuators and sensors outside of the hand skeleton. Then, along with the tendon-driven actuation, a finger posture information is to be obtained with a flex sensor, whose relation to the joint rotation needs to be defined. Regarding this issue, previous research modeled a linear relation between the flex sensor reading and the rotation angle (Saggio, 2012). Also, the relation between the distal and peripheral interphalangeal joint angles are found to be modeled with a polynomial equation (Yang et al., 2016). Then, unless MCP and PIP are independently controlled, only one flex sensor can be used to estimate overall finger posture by collecting posture-flex sensor output data. Otherwise, separate flex sensors need to be installed for the MCP joint and the PIP/DIP joint group. Then, the fingertip position can be estimated and controlled with a feedback system.

**Figure 6:**
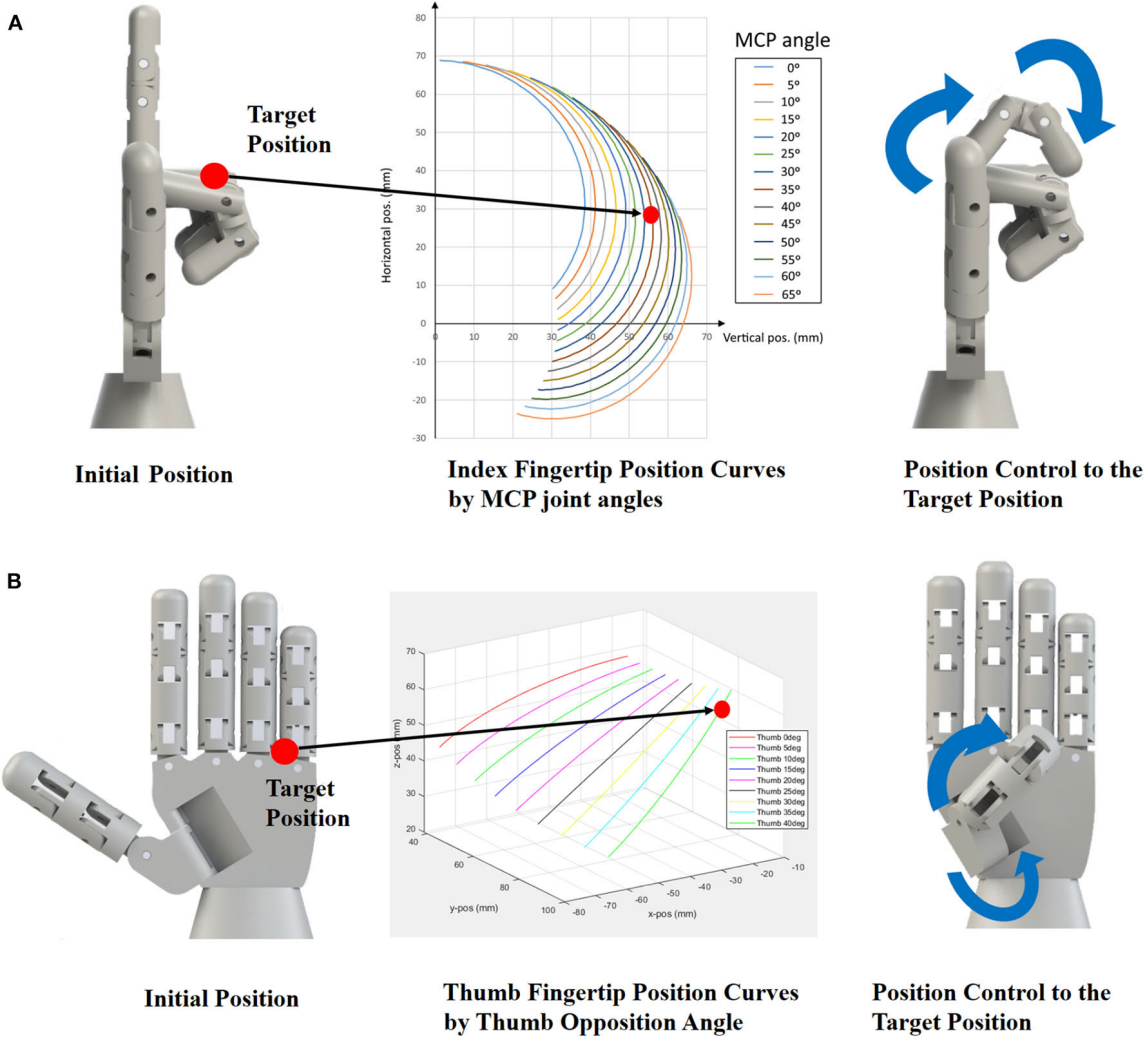
Finger posture mapping based on the Cartesian space mapping. Given the target position of the fingertip (red circle), an end-effector position curve closest to the position is selected. **(A)** Control of the index finger posture. **(B)** Control of the thumb posture.

For the control of the robotic hand posture, we adopted a synergistic control approach (Salvietti, 2018). We controlled the posture of each finger to locate the end-effector (fingertip) to the desired position. This approach is called the Cartesian space mapping (Ficuciello et al., 2018). Figure 6 shows the control strategies of the index finger and the thumb. For the index finger control, we fixed the MCP joint angle and collected the end-effector position by rotating the PIP joint. The data was collected for the MCP angles ranging from 0 to 65 degrees. The rotation angle of the PIP and MCP joint angles can be tracked and controlled with the flex sensor. Then, the effector position is one-to-one mapped to the flex sensor reading values. Then, given the target position for the index finger, a closest point on the end-effector position curve is selected (Figure 6A). The index finger pose is changed for the target position by controlling the PIP and MCP joint angles mapped to flex sensor readings. Similarly, for the thumb control, the end-effector position by the thumb opposition angle is collected by rotating the thumb's IP joint. The data was collected for the thumb opposition angles ranging from 0 to 40 degrees. Then, given the target position for the thumb, the closest point on the end-effector position curve is selected (Figure 6B). The thumb pose is changed for the target position by controlling the IP joint, and thumb opposition angles mapped to flex sensor readings. For other fingers, we implemented only extrinsic finger muscle movement. Thus, only one end-effector trajectory could be collected, which was used for the finger pose control.

### 2.3. Tactile Sensor

**Figure 7:**
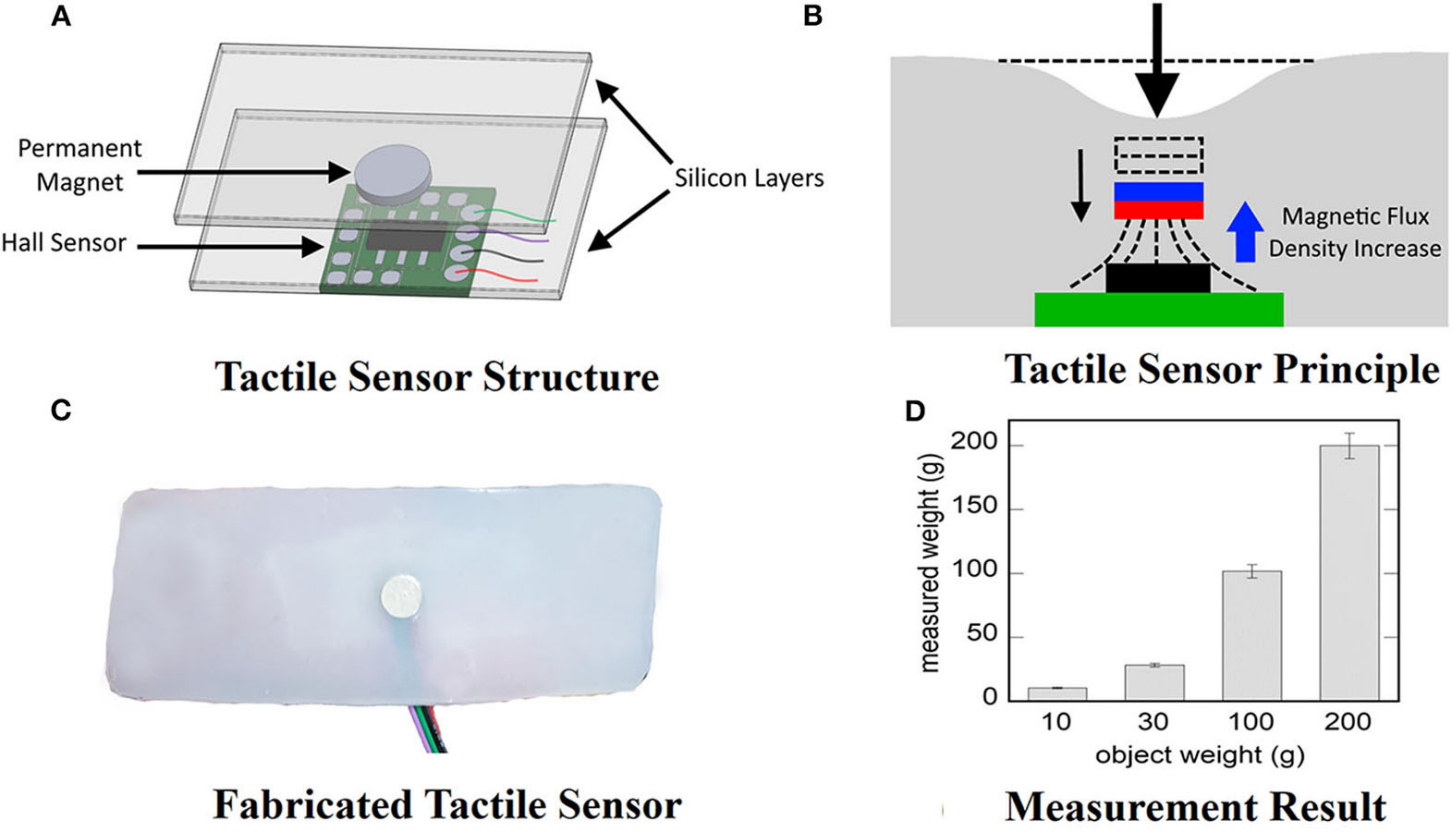
Tactile sensor to manipulate an object with the robotic hand. **(A)** Tactile sensor structure. **(B)** Tactile sensor working principle. If the surface is pressed down, the permanent magnet moves closer to the hole sensor, increasing the magnetic flux density. **(C)** Photo of a fabricated tactile sensor. **(D)** The measured weight of reference objects. Error bars indicate standard errors.

We built a tactile sensor to manipulate an object with the robotic hand actuated with our proposed exo-glove. Figure 7 shows the tactile sensor for the robot hand manipulation. Figure 7A shows the structure of the tactile sensor (Wang et al., 2016). It consists of a permanent magnet, a 3-DOF hall sensor (MLX90393, Melexis, Ieper, Belgium), and silicon layers (EcoFlex0030, Smooth-on, PA, U.S.A.) enclosing the sensor and magnet. We fabricated the sensor by first creating a silicon layer with a mold, placing the permanent magnet, and creating another silicon layer. Finally, the hall sensor was attached to the top. Figure 7B shows the working principle of the sensor. Once the outer layer silicon is pressed, the permanent magnet moves closer to the hall sensor, which increases the magnetic flux. Thus, the magnetic field strength read by the sensor is increased, signaling increased contact force. Figure 7C shows the fabricated sensor sample. The sensor is worn around the robotic hand's fingertips and senses the contact at the fingertip. To evaluate the fabricated sensor's precision, we measured the weight of four reference objects (10, 30, 100, and 200 g) 10 times. Figure 7D shows the mean measured weight for the reference objects with standard error.

## 3. Performance Evaluation

We first test the functionality of the robotic hand actuated with the exo-glove with standard grasp performance tests (Falco et al., 2015). Then, robotic hand with exo-glove was evaluated in terms of position repeatability, and object grasping ability.

### 3.1. Standard Grasp Performance Test

A recent trend in the robotic community is to standardize the performance test for particular applications and tasks (Bonsignorio et al., 2014). Regarding a robotic hand's grasping performance, Falco et al. proposed several performance test methods to evaluate the robotic system (Falco et al., 2015). Among various test methods, we chose three tests for touch sensitivity, finger strength, and grasp strength.

#### 3.1.1. Touch Sensitivity

**Figure 8:**
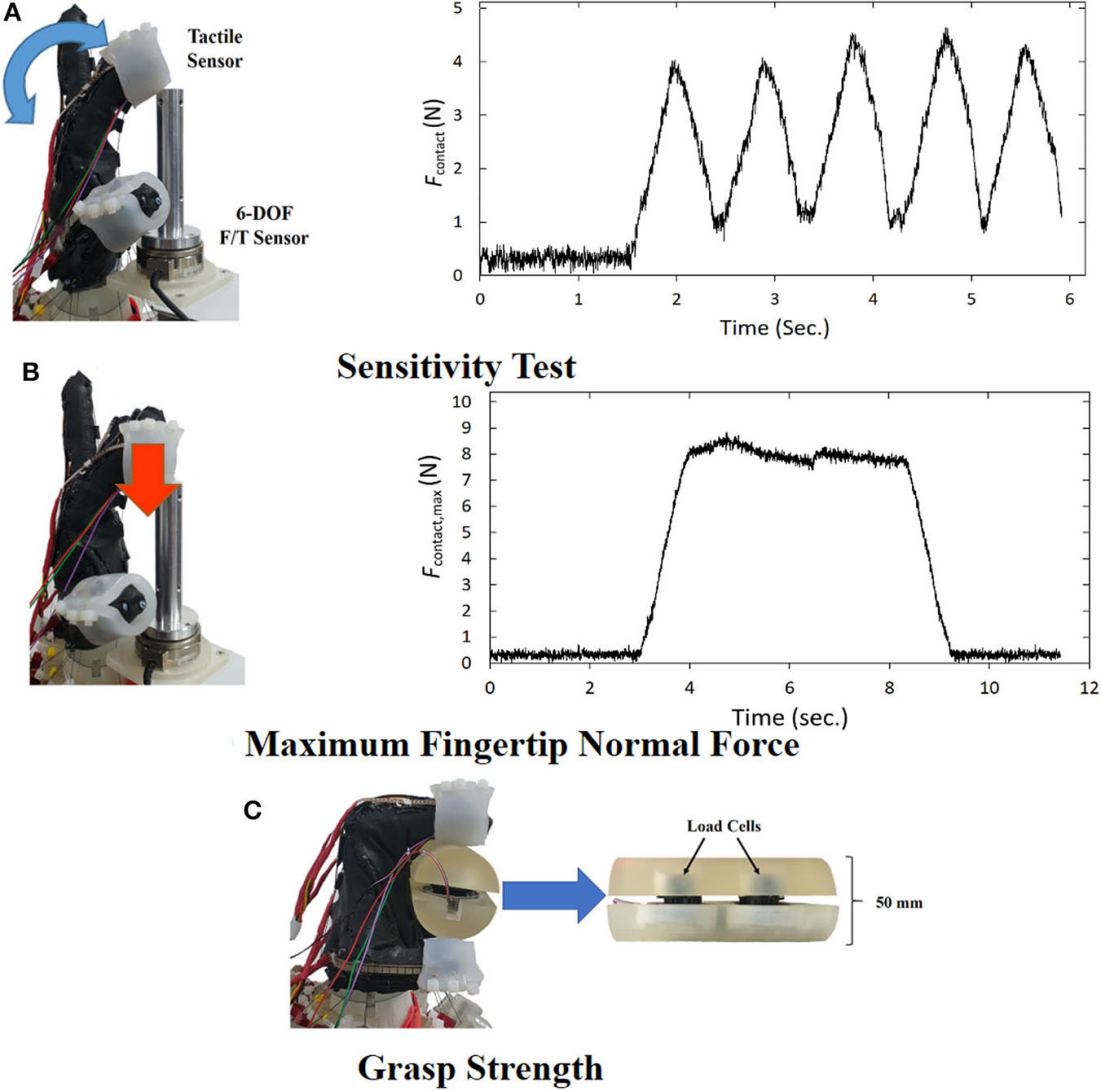
Three standard grasp performance tests. **(A)** Sensitivity test setup (left) and the contact force measurement result. **(B)** Maximum fingertip contact force measurement setup (left) and the force measurement result (right) **(C)** Grasp measurement setup with the custom-built grasp force measurement device.

Falco et al. suggested measuring the contact during a dynamic test to evaluate robotic finger sensitivity (Falco et al., 2015). The test is implemented by repeating motion with three phases, moving a robotic finger to a cylinder attached to a 6-DOF force/torque sensor (Mini45, ATI Industrial Automation, Apex, NC, U.S.A), and retracting the finger from the object (Figure 8A, left). To detect the contact at the fingertip, we used the tactile sensor described in section 2.3. We controlled the finger rotation speed to 50 °/*s* and the contact force was recorded at the rate of 1 kHz. Figure 8A (right) shows the measured contact force *F*_*contact*_ computed by the *L*_2_ norm. The mean of the maximum contact force *F*_*cotnact*_ was 4.2 ± 0.30 for five repetitions of the motion. The mean *F*_*contact*_ is significantly lower than those of the same test conducted by Falco et al., which ranged from 10 to 30 N.

#### 3.1.2. Fingertip Maximum Contact Force

We measured the finger strength by exerting the highest force at the fingertip and measuring the force with the 6-DOF force/torque sensor used for the touch sensitivity test, as shown in Figure 8B (left). The robotic hand's finger pressed down the cylindrical object attached to the force/torque sensor and the contact force was recorded at the rate of 1 kHz. Figure 8A (right) shows the measured contact force *F*_*contact*_ computed by the *L*_2_ norm. The mean of the fingertip maximum contact force for five trials is 8.84 ± 0.23, which is around the range of the fingertip maximum force of Hand 1's finger 2 in Falco et al. (2015).

#### 3.1.3. Grasp Strength

The grasp strength of the robotic hand was evaluated by measuring the maximum grasp strength of wrapping a cylindrical device proposed by Falco et al. (2015). We fabricated a 50-mm diameter split cylinder with a pair of 1-DOF load cells for determining the grasp force, as suggested by the reference (Figure 8C). The test was conducted by grasping the measurement device with the robotic hand at the maximum grasp force. The mean of the maximum grasp force for five trials is 30.49 ± 2.19 N, which is significantly lower than those measured by Falco et al.'s reference. A possible explanation for the lower grasp force can be found in the asymmetry of the finger configuration of our proposed anthropomorphic hand. In contrast, the robotic hands used in the reference had the symmetric finger configuration. That is more efficient to press down the cylindrical shape in the normal direction of the fingers than an anthropomorphic hand.

### 3.2. Object Grasping Test

In this section, we evaluate the robot hand's ability to mimic human grasping motion. Our proposed design can independently move five fingers, and emulate the functionalities of the thumb's thenar muscles. We conducted the Cutkosky taxonomy, which is proposed to evaluate a hand's performance for manufacturing tasks. The taxonomy proposes total 16 grasping poses, largely classified as power and precision grasps.

**Figure 9:**
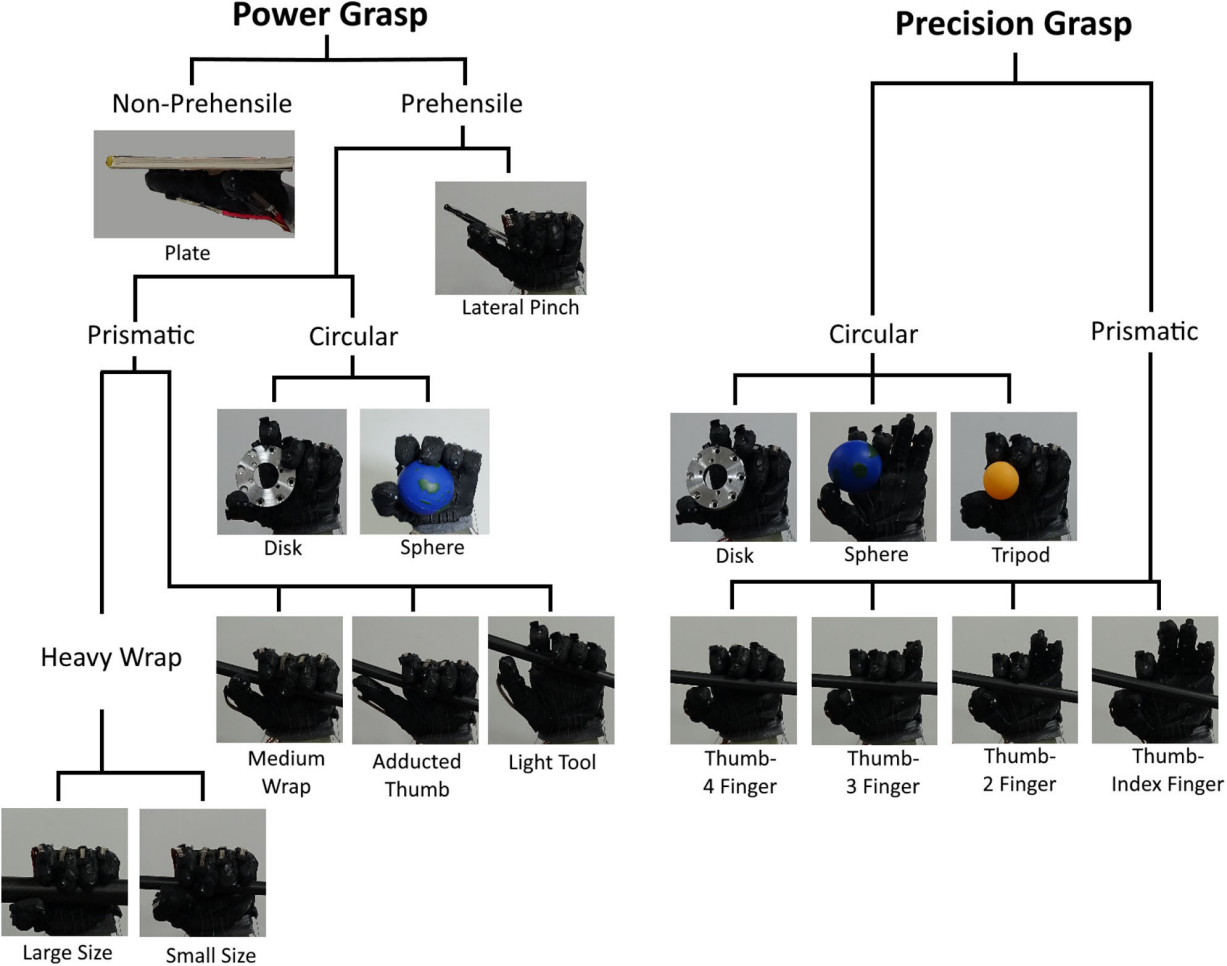
The hand poses realized by our robotic hand, according to the Cutkosky taxonomy. The hand was able to realize all 16 poses proposed by the taxonomy.

Figure 9 shows the results of the Cutkosky taxonomy test. Our proposed robotic hand was able to perform all 16 poses proposed in the Cukosky taxonomy. This is mainly because our proposed design has actuators outside of the hand, enabling relatively complex hand motion, including the motion of the little finger and thenar muscles. Since our design can potentially include more actuators, the complexity of the hand motion, e.g., intrinsic muscles of each finger, can be increased.

### 3.3. Fingertip Position Trajectories

**Figure 10:**
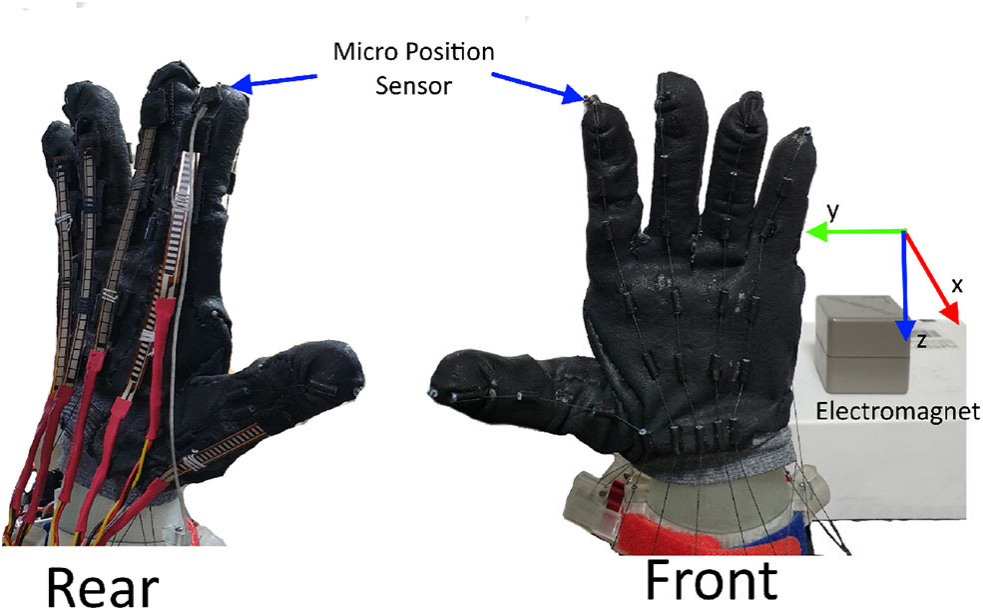
Fingertip motion measurement setup. An high-resolution position sensor is attached to the fingertip. The position was recorded as a relative distance from the AC electromagnet located behind the robotic hand.

In this subsection, we test our proposed robot hand's ability to reliably strike finger position using only flex sensor information. Each finger was programmed to repeat the flexion and extension motions ten times. To measure the position of the fingertip, we used a high-resolution electromagnetic sensor (Liberty, Polhemus, VM, U.S.A.). Figure 10 the nominal position resolution of the sensor was 0.005 mm within the range of 24 inches.

**Figure 11:**
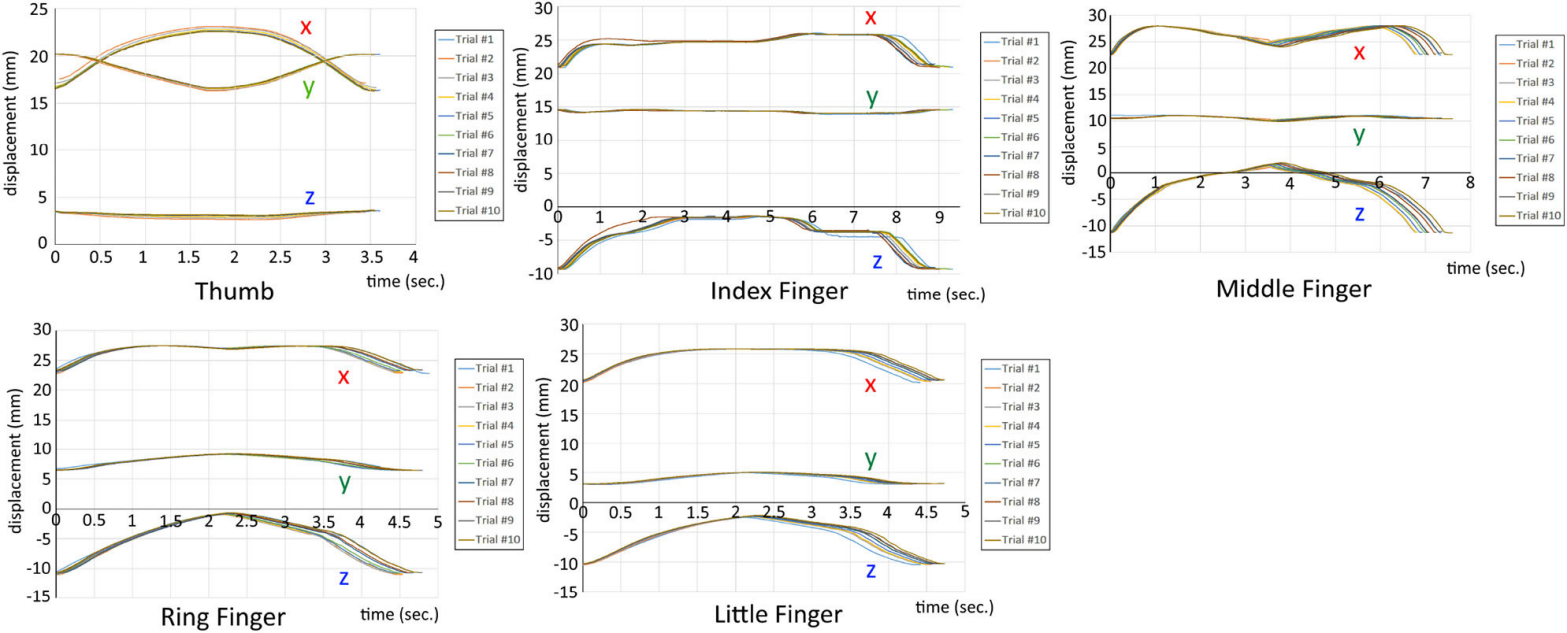
The measurement data of fingertip position for 10 times of flexion/extension motion. The data was sampled at the frequency of 240 Hz.

Figure 11 shows the tracking of five fingers and show that the motions are repeatable with only flex sensor input and control. The profile of index finger's z-direction (vertical direction) differs from other fingers since it has an intrinsic MCP flexion/extension tendons and thus has discontinuities in the flexion routine. To evaluate the accuracy of the repeated motion for the five fingers, we calculated the Euclidean distance of the fingertip position from the electromagnet at the peak displacements. We then calculated the standard deviation of the Euclidean distance at the ten peak displacements as a measure of accurate repetition. Table 1 shows the standard deviation for the five fingers, which is less than 0.15 cm except for the middle finger. A possible explanation for the relatively large standard deviation for the middle finger is the loose attachment of the glove to the skeleton.

**Table 1:** Standard deviation of the peak displacement for the five finger position measurement in Figure 11.

	**Thumb**	**Index finger**	**Middle finger**	**Ring finger**	**Little finger**
Standard dev. (cm)	0.112	0.014	0.522	0.072	0.01

### 3.4. Object Gripping Test With the Precision Grip

In the last experiment, we evaluate the robot hand's grip in various aspects. A typical robotic gripper is designed to meet the constraints required for the task, such as a higher payload. One of the recent approaches to increase a robot gripper's payload is to introduce the variable stiffness of a joint. The method often contracts the tendon so that the stiffness of the joint is increased. Noting this, we test the possibility of increasing the gripping payload by increasing the stiffness of a finger joint, as well as measuring normal payload by increasing the gripping force.

**Figure 12:**
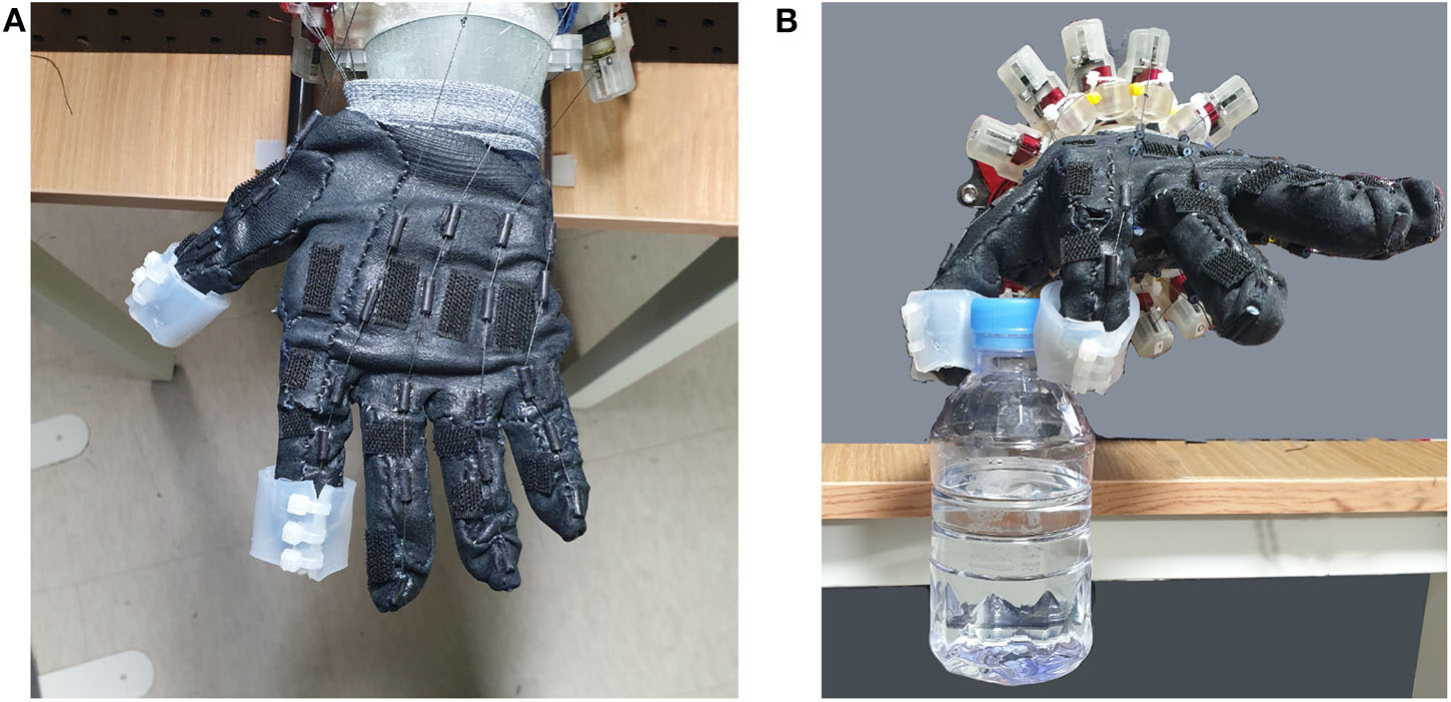
Object gripping task with the precision grip. **(A)** The fingertips of the thumb and the index finger were covered with the silicone skin. **(B)** The robotic hand grasped a PET water bottle with a precision grip.

**Figure 13:**
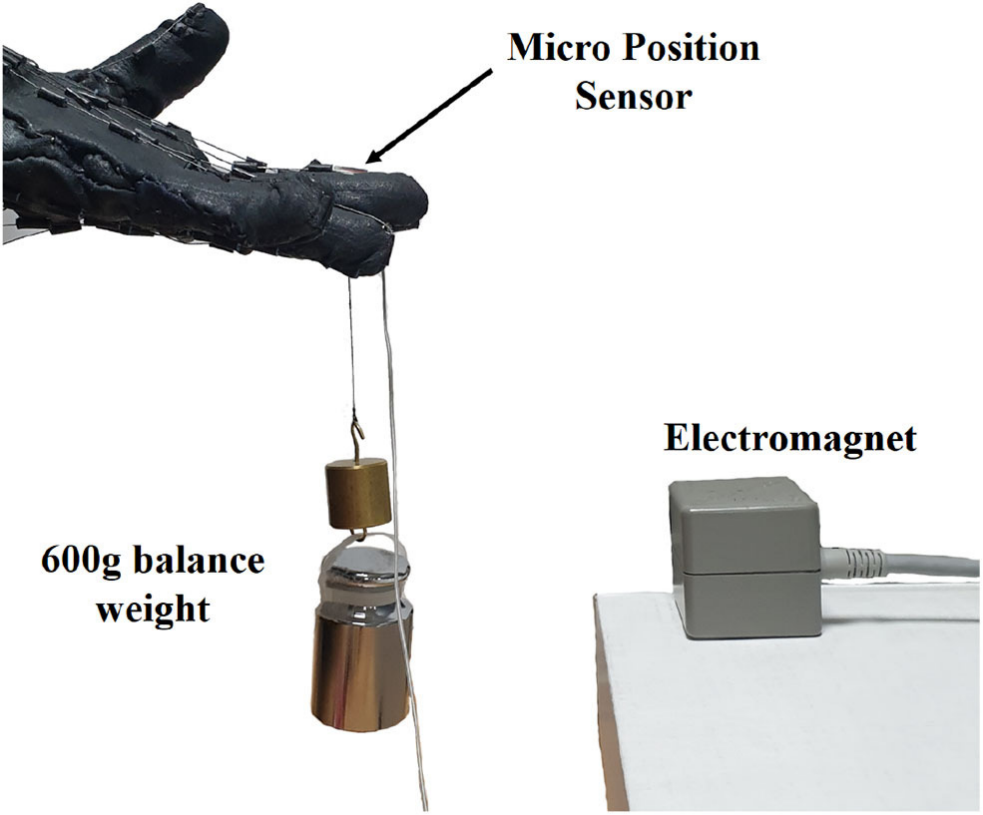
Measurement setup for the nominal stiffness of the three conditions—low, high, and high-torque precision grip conditions. A high-resolution electromagnetic sensor attached to the index fingertip measured the displacement of the fingertip by 600 g of balance-weight.

We chose the precision grip for the object grasping task, considering that it is one of the most common object grasping strategies. To mimic the human skin for the gripping task, we fabricated pieces of silicone skin with EcoFlex0030 (Smooth-on, PA, U.S.A.). Prior to the test, the thumb and index fingertip were covered with the silicone skin, as shown in Figure 12. We measured the maximum payload of the robot hand under three conditions. Under the low stiffness condition, the thumb and index's finger's extensor tendons were loosened during the grasp task. No torque was applied to the fingers once the hand firmly grasped the object. Under the high stiffness condition, the extensor tendons were activated until the hand firmly grasped the object. Under the high-torque precision grip condition, the torque, thus normal force is applied to the fingers even after grasping the object. The nominal torque was 1.40kg·cm. Before the object gripping test, we measured the nominal stiffness of the three condition with the experiment setup in Figure 13. A high-resolution electromagnetic magnetic sensor recorded the position displacement of the fingertip due to 600 g of balance-weight. Then, the nominal stiffness for the three conditions was calculated from the displacement as 0.35 ± 0.13, 0.58 ± 0.1, and 2.17 ± 0.89 N/mm for low, high, and high-torque precision grip conditions, respectively.

Table 2 shows the results of the test, as expected, increasing the tendon stiffness increased the maximum payload of the fingertip. However, regardless of the increased stiffness, the maximum payload was higher when the grasping force was maintained after grasping the object. The result can be explained with the Coulombic friction, where the lateral friction is increased with the increase of the normal directional force.

**Table 2:** The result of the gripping test under three conditions.

**Condition**	**Low stiffness**	**High stiffness**	**High-torque precision grip**
Max payload (g)	140	224	420

## 4. Conclusion

In this paper, we proposed a novel robot hand exo-glove that was designed to be combined with a robot hand skeleton to form a robotic hand. The design of our robot exo-glove platform can be applied to a prosthetic hand with more motion functionality and less dimension than previous works, e.g., Rudd et al. (2019). The proposed platform has the versatility of hand/finger shape, finger length, and even the number of fingers. Thus, it can significantly enhance the flexibility of a wearable robotic hand. Additional fingertip sensors can enable our glove to bend by the user's intention. Therefore, the device's dimension will be much smaller than the previous efforts requiring fingertip sensors, such as Al-Fahaam et al. (2018).

The results of the evaluation indicate that the glove-worn robot hand can strike all the poses proposed by Cutkosky (1989). Also, the fingertip position tracking data shows that the finger position control can be repeated with no significant error. Finally, we demonstrated that increasing fingertip stiffness could enhance the grip force when no gripping force is applied after the grip event. Overall, we demonstrated that our proposed robot hand with exo-glove could be a viable robot hand platform for variable manual tasks. In our future work, we will continue to improve the robot hand platform by efficiently using rigid and soft structures. Also, we are planning to incorporate a flexible touch sensor for the robot hand to sense objects and conduct manual tasks effectively.

The future work will extend the exo-glove to applications such as prosthetic hand and power assist glove. Especially, the application to the prosthetic hand will require the further implementation of the interface for intention cognition. We will conduct additional evaluations of the exo-glove in terms of device manipulability. We are also planning to apply lighter actuators to enhance the wearability of the exo-glove.

## Data Availability Statement

All datasets generated for this study are included in the article/supplementary material.

## Author Contributions

JP and WL conceived the research idea and analyzed the results JP and IH setup the system concept and implemented the hardware. JP prepared for the experiment. All authors reviewed the manuscript.

## Conflict of Interest

The authors declare that the research was conducted in the absence of any commercial or financial relationships that could be construed as a potential conflict of interest.
